# 2-Bromo-3-phenyl-1-(3-phenyl­sydnon-4-yl)prop-2-en-1-one

**DOI:** 10.1107/S1600536810015205

**Published:** 2010-04-30

**Authors:** Jia Hao Goh, Hoong-Kun Fun, Nithinchandra, B. Kalluraya

**Affiliations:** a X-ray Crystallography Unit, School of Physics, Universiti Sains Malaysia, 11800 USM, Penang, Malaysia; b Department of Studies in Chemistry, Mangalore University, Mangalagangotri, Mangalore 574 199, India

## Abstract

The title sydnone derivative [systematic name: 2-bromo-1-(5-oxido-3-phenyl-1,2,3-oxadiazo­lium-4-yl)-3-phenyl­prop-2-en-1-one], C_17_H_11_BrN_2_O_3_, exists in a *Z* configuration with respect to the acyclic C=C bond. An intra­molecular C—H⋯Br hydrogen bond generates a six-membered ring, producing an *S*(6) ring motif. The 1,2,3-oxadiazole ring in the sydnone unit is essentially planar [maximum deviation = 0.011 (2) Å] and forms dihedral angles of 55.39 (13) and 57.12 (12)° with the two benzene rings. In the crystal structure, inter­molecular C—H⋯O hydrogen bonds link mol­ecules into two-mol­ecule-thick arrays parallel to the *bc* plane. The crystal structure also features a short inter­molecular N⋯C contacts [3.030 (3) Å] as well as C—H⋯π and π–π inter­actions [centroid–centroid distances = 3.3798 (11) and 3.2403 (12) Å].

## Related literature

For general background to and applications of sydnone deriv­atives, see: Baker *et al.* (1949 ▶); Hedge *et al.* (2008 ▶); Rai *et al.* (2008 ▶). For related structures, see: Baker & Ollis (1957 ▶); Goh *et al.* (2010 ▶); Grossie *et al.* (2009 ▶). For graph-set descriptions of hydrogen-bond ring motifs, see: Bernstein *et al.* (1995 ▶). For the stability of the temperature controller used for the data collection, see: Cosier & Glazer (1986 ▶).
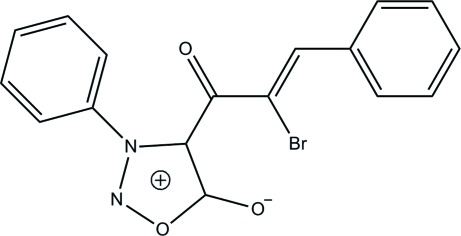


## Experimental

### 

#### Crystal data

C_17_H_11_BrN_2_O_3_
*M* *_r_* = 371.19
Monoclinic, 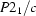

*a* = 15.0512 (5) Å
*b* = 5.9887 (2) Å
*c* = 22.3940 (6) Å
β = 129.444 (2)°
*V* = 1558.80 (8) Å^3^
*Z* = 4
Mo *K*α radiation
μ = 2.65 mm^−1^
*T* = 293 K
0.38 × 0.27 × 0.17 mm

#### Data collection

Bruker SMART APEXII CCD area-detector diffractometer
Absorption correction: multi-scan (*SADABS*; Bruker, 2009 ▶) *T*_min_ = 0.435, *T*_max_ = 0.658
18185 measured reflections
4816 independent reflections
3232 reflections with *I* > 2σ(*I*)
*R*_int_ = 0.030

#### Refinement

*R*[*F* ^2^ > 2σ(*F* ^2^)] = 0.041
*wR*(*F* ^2^) = 0.103
*S* = 1.02
4816 reflections
252 parameters
All H-atom parameters refined
Δρ_max_ = 0.64 e Å^−3^
Δρ_min_ = −0.83 e Å^−3^

### 

Data collection: *APEX2* (Bruker, 2009 ▶); cell refinement: *SAINT* (Bruker, 2009 ▶); data reduction: *SAINT*; program(s) used to solve structure: *SHELXTL* (Sheldrick, 2008 ▶); program(s) used to refine structure: *SHELXTL*; molecular graphics: *SHELXTL*; software used to prepare material for publication: *SHELXTL* and *PLATON* (Spek, 2009 ▶).

## Supplementary Material

Crystal structure: contains datablocks global, I. DOI: 10.1107/S1600536810015205/tk2656sup1.cif

Structure factors: contains datablocks I. DOI: 10.1107/S1600536810015205/tk2656Isup2.hkl

Additional supplementary materials:  crystallographic information; 3D view; checkCIF report

## Figures and Tables

**Table 1:** Hydrogen-bond geometry (Å, °) *Cg*1 is the centroid of the C12–C17 benzene ring.

*D*—H⋯*A*	*D*—H	H⋯*A*	*D*⋯*A*	*D*—H⋯*A*
C2—H2*A*⋯O3^i^	0.87 (3)	2.58 (3)	3.126 (3)	122 (2)
C3—H3*A*⋯O3^i^	0.93 (3)	2.53 (3)	3.140 (4)	124 (2)
C5—H5*A*⋯O2^ii^	0.92 (3)	2.47 (3)	3.388 (3)	171 (2)
C17—H17*A*⋯Br1	0.97 (3)	2.66 (3)	3.364 (3)	130 (3)
C14—H14*A*⋯*Cg*1^iii^	0.96 (3)	2.86 (3)	3.639 (3)	139 (2)

Symmetry codes: (i) 
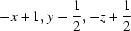
; (ii) 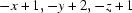; (iii) 
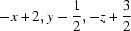
.

**Fig. 1.**
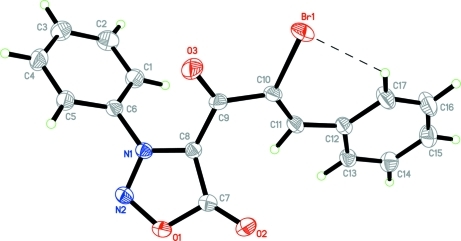
The molecular structure of the title compound, showing 30% probability displacement ellipsoids for non-H atoms and the atom-numbering scheme.

**Fig. 2.**
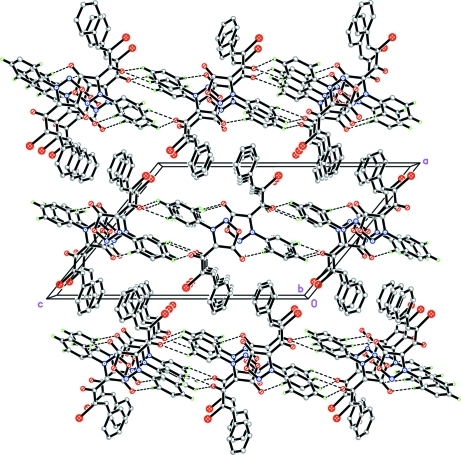
The crystal structure of the title compound, viewed along the b axis, showing two-molecule-thick arrays parallel to the bc plane. Hydrogen atoms not involved in intermolecular interactions (dashed lines) have been omitted for clarity.

## supplementary crystallographic information

### Comment

Sydnones constitute a well-defined class of mesoionic compounds consisting of 1,2,3-oxadiazole ring system. The introduction of the concept of mesoionic structure for certain heterocyclic compounds in 1949 has proved to be fruitful development in heterocyclic chemistry (Baker *et al.*, 1949). The study of sydnones still remains a field of interest because of their electronic structure and also because of the various types of biological activities displayed by some of them. Interest in sydnone derivatives has also been encouraged by the discovery that they exhibit various pharmacological activities (Hedge *et al.*, 2008; Rai *et al.*, 2008). Chalcone derivatives were obtained by the base catalyzed condensation of 4-acetyl-3-aryl sydnones with aromatic aldehydes in aqueous alcoholic medium at 273–278 K. Bromination of chalcones with bromine in glacial acetic acid afforded dibromo chalcones. The dibromochalcones obtained were subjected to dehydrobromination in presence of triethylamine in dry benzene medium to get 2-bromopropenones.

The title sydnone derivative (Fig. 1) exists in a *Z* configuration with respect to the acyclic C10═C11 bond [C10═C11 = 1.335 (3) Å; torsion angle of C9–C10–C11–C12 = 179.9 (2)°]. An intramolecular C17—H17A···Br1 hydrogen bond (Table 1) generates a six-membered ring, producing an *S*(6) ring motif (Bernstein *et al.*, 1995). The 1,2,3-oxadiazole ring (N1/N2/O1/C7/C8) is essentially planar, with the maximum deviation of -0.011 (2) Å at atom N2. The C1-C6 and C12-C17 benzene rings incline at dihedral angles of 55.39 (13) and 57.12 (12)°, respectively, to the 1,2,3-oxadiazole ring. As reported previously (Goh *et al.*, 2010; Grossie *et al.*, 2009), the exocyclic C7–O2 bond length of 1.193 (3) Å does not support the formulation of Baker & Ollis (1957), which reported the delocalization of a positive charge in the ring, and a negative charge in the exocyclic oxygen. The bond lengths are comparable to those observed in closely related sydnone structures (Goh *et al.*, 2010; Grossie *et al.*, 2009).

In the crystal structure, intermolecular C2—H2A···O3, C3—H3A···O3 and C5—H5A···O2 hydrogen bonds (Table 1) link molecules into two-molecule-thick arrays parallel to the *bc* plane (Fig. 2). An interesting feature of the crystal structure is the presence of a short N2···C7 interaction [N2···C7 = 3.030 (3) Å; symmetry code: -x+1, -y+1, -z+1] which is shorter than the sum of the van der Waals radii of the relevant atoms. The crystal structure is further stabilized by weak C14—H14A···*Cg*1 (Table 1) as well as aromatic *Cg*2···*Cg*2 stacking interactions [*Cg*2···*Cg*2^ii^ = 3.3798 (11) Å and *Cg*2···*Cg*2^iii^ = 3.2403 (12) Å; (ii) -x+1, -y+2, -z+1 and (iii) -x+1, -y+1, -z+1 where *Cg*1 and *Cg*2 are the centroids of C12-C17 benzene and 1,2,3-oxadiazole rings, respectively].

### Experimental

To a stirred suspension of 2,3-dibromo-1-(3'-phenylsydnon-4'-yl)-3-phenyl-propan-1-one (0.01 mol) in benzene was added a solution of triethylamine (0.04 mol) in dry benzene (20 ml) at room temperature. The reaction mixture was stirred at room temperature for 24 h. After removing the separated triethylamine hydrobromide, the filtrate was concentrated under reduced pressure to give 2-bromo propenone which was purified by recrystallization from ethanol. Single crystals suitable for X-ray analysis were obtained from a 1:2 mixture of DMF and ethanol by slow evaporation.

### Refinement

All hydrogen atoms were located from difference Fourier map [range of C—H = 0.87 (3)–0.97 (3) Å] and allowed to refine freely.

### Crystal data

**Table:**

C_17_H_11_BrN_2_O_3_	*F*(000) = 744
*M**_r_* = 371.19	*D*_x_ = 1.582 Mg m^−^^3^
Monoclinic, *P*2_1_/*c*	Mo *K*α radiation, λ = 0.71073 Å
Hall symbol: -P 2ybc	Cell parameters from 4335 reflections
*a* = 15.0512 (5) Å	θ = 2.4–29.8°
*b* = 5.9887 (2) Å	µ = 2.65 mm^−^^1^
*c* = 22.3940 (6) Å	*T* = 293 K
β = 129.444 (2)°	Block, yellow
*V* = 1558.80 (8) Å^3^	0.38 × 0.27 × 0.17 mm
*Z* = 4	

### Data collection

**Table:**

Bruker SMART APEXII CCD area-detector diffractometer	4816 independent reflections
Radiation source: fine-focus sealed tube	3232 reflections with *I* > 2σ(*I*)
graphite	*R*_int_ = 0.030
φ and ω scans	θ_max_ = 30.6°, θ_min_ = 1.8°
Absorption correction: multi-scan (*SADABS*; Bruker, 2009)	*h* = −20→21
*T*_min_ = 0.435, *T*_max_ = 0.658	*k* = −8→8
18185 measured reflections	*l* = −32→29

### Refinement

**Table:**

Refinement on *F*^2^	Primary atom site location: structure-invariant direct methods
Least-squares matrix: full	Secondary atom site location: difference Fourier map
*R*[*F*^2^ > 2σ(*F*^2^)] = 0.041	Hydrogen site location: inferred from neighbouring sites
*wR*(*F*^2^) = 0.103	All H-atom parameters refined
*S* = 1.02	*w* = 1/[σ^2^(*F*_o_^2^) + (0.043*P*)^2^ + 0.6594*P*] where *P* = (*F*_o_^2^ + 2*F*_c_^2^)/3
4816 reflections	(Δ/σ)_max_ = 0.001
252 parameters	Δρ_max_ = 0.64 e Å^−^^3^
0 restraints	Δρ_min_ = −0.83 e Å^−^^3^

### Special details

**Table:**

Geometry. All esds (except the esd in the dihedral angle between two l.s. planes) are estimated using the full covariance matrix. The cell esds are taken into account individually in the estimation of esds in distances, angles and torsion angles; correlations between esds in cell parameters are only used when they are defined by crystal symmetry. An approximate (isotropic) treatment of cell esds is used for estimating esds involving l.s. planes.
Refinement. Refinement of F^2^ against ALL reflections. The weighted R-factor wR and goodness of fit S are based on F^2^, conventional R-factors R are based on F, with F set to zero for negative F^2^. The threshold expression of F^2^ > 2sigma(F^2^) is used only for calculating R-factors(gt) etc. and is not relevant to the choice of reflections for refinement. R-factors based on F^2^ are statistically about twice as large as those based on F, and R- factors based on ALL data will be even larger.

### Fractional atomic coordinates and isotropic or equivalent isotropic displacement parameters (Å^2^)

**Table:**

	*x*	*y*	*z*	*U*_iso_*/*U*_eq_	
Br1	0.90010 (2)	0.71291 (5)	0.499676 (17)	0.06462 (12)	
O1	0.50418 (13)	0.7541 (2)	0.52358 (8)	0.0376 (3)	
O2	0.69784 (14)	0.7260 (2)	0.61954 (8)	0.0452 (4)	
O3	0.66790 (15)	0.8945 (4)	0.42133 (10)	0.0688 (5)	
N1	0.49069 (14)	0.7454 (2)	0.42344 (9)	0.0320 (3)	
N2	0.42695 (15)	0.7565 (3)	0.44402 (10)	0.0370 (4)	
C1	0.4535 (2)	0.5529 (4)	0.31430 (14)	0.0492 (5)	
C2	0.3975 (2)	0.5418 (5)	0.23612 (15)	0.0590 (7)	
C3	0.3218 (2)	0.7063 (5)	0.18686 (14)	0.0584 (7)	
C4	0.3014 (2)	0.8844 (5)	0.21526 (14)	0.0603 (7)	
C5	0.3563 (2)	0.9003 (4)	0.29352 (13)	0.0485 (5)	
C6	0.43174 (17)	0.7337 (3)	0.34129 (11)	0.0364 (4)	
C7	0.61978 (17)	0.7329 (3)	0.55169 (12)	0.0345 (4)	
C8	0.60568 (16)	0.7289 (3)	0.48251 (11)	0.0331 (4)	
C9	0.68943 (17)	0.7596 (3)	0.46933 (12)	0.0394 (4)	
C10	0.79547 (16)	0.6228 (4)	0.51474 (11)	0.0379 (4)	
C11	0.80979 (17)	0.4513 (4)	0.55817 (12)	0.0387 (4)	
C12	0.90186 (17)	0.2897 (3)	0.60789 (12)	0.0399 (4)	
C13	0.87661 (19)	0.1127 (4)	0.63547 (13)	0.0437 (5)	
C14	0.9577 (2)	−0.0474 (4)	0.68386 (14)	0.0520 (6)	
C15	1.0664 (2)	−0.0325 (5)	0.70620 (16)	0.0600 (7)	
C16	1.0933 (2)	0.1406 (5)	0.68038 (19)	0.0679 (8)	
C17	1.0137 (2)	0.3022 (5)	0.63199 (17)	0.0588 (7)	
H1A	0.505 (2)	0.445 (4)	0.3489 (14)	0.054 (7)*	
H2A	0.411 (2)	0.426 (5)	0.2196 (16)	0.065 (8)*	
H3A	0.286 (2)	0.695 (4)	0.1345 (17)	0.059 (8)*	
H4A	0.251 (2)	0.995 (5)	0.1826 (16)	0.072 (9)*	
H5A	0.343 (2)	1.015 (4)	0.3145 (14)	0.053 (7)*	
H11A	0.7463 (18)	0.430 (4)	0.5558 (12)	0.039 (6)*	
H13A	0.802 (2)	0.111 (4)	0.6204 (13)	0.045 (6)*	
H14A	0.937 (2)	−0.169 (5)	0.7008 (16)	0.064 (8)*	
H15A	1.122 (3)	−0.135 (5)	0.7404 (17)	0.074 (9)*	
H16A	1.167 (3)	0.148 (6)	0.6941 (19)	0.086 (10)*	
H17A	1.034 (2)	0.422 (5)	0.6138 (16)	0.073 (9)*	

### Atomic displacement parameters (Å^2^)

**Table:**

	*U*^11^	*U*^22^	*U*^33^	*U*^12^	*U*^13^	*U*^23^
Br1	0.05352 (16)	0.0904 (2)	0.0739 (2)	0.00434 (13)	0.05173 (15)	0.01659 (14)
O1	0.0456 (8)	0.0357 (7)	0.0432 (8)	0.0004 (6)	0.0337 (7)	−0.0005 (6)
O2	0.0507 (9)	0.0494 (9)	0.0344 (7)	0.0007 (7)	0.0265 (7)	0.0009 (6)
O3	0.0518 (9)	0.0982 (14)	0.0619 (11)	0.0083 (10)	0.0387 (9)	0.0395 (10)
N1	0.0357 (8)	0.0292 (8)	0.0359 (8)	0.0006 (6)	0.0250 (7)	−0.0002 (6)
N2	0.0399 (8)	0.0339 (8)	0.0432 (9)	0.0009 (6)	0.0293 (8)	−0.0012 (7)
C1	0.0559 (13)	0.0462 (12)	0.0469 (12)	0.0092 (11)	0.0333 (11)	−0.0010 (10)
C2	0.0718 (17)	0.0574 (15)	0.0522 (14)	0.0037 (13)	0.0415 (13)	−0.0145 (12)
C3	0.0565 (14)	0.0771 (18)	0.0352 (12)	0.0021 (13)	0.0261 (11)	−0.0098 (12)
C4	0.0563 (14)	0.0746 (18)	0.0367 (12)	0.0199 (13)	0.0233 (11)	0.0045 (12)
C5	0.0504 (12)	0.0527 (13)	0.0381 (11)	0.0135 (10)	0.0261 (10)	−0.0010 (10)
C6	0.0348 (9)	0.0411 (10)	0.0335 (9)	−0.0005 (8)	0.0218 (8)	−0.0036 (8)
C7	0.0411 (10)	0.0282 (9)	0.0401 (10)	−0.0015 (7)	0.0285 (9)	0.0002 (8)
C8	0.0350 (9)	0.0327 (9)	0.0340 (9)	0.0010 (7)	0.0230 (8)	0.0024 (7)
C9	0.0378 (10)	0.0495 (12)	0.0352 (10)	−0.0033 (8)	0.0252 (9)	0.0032 (9)
C10	0.0345 (9)	0.0492 (11)	0.0377 (10)	−0.0044 (8)	0.0265 (8)	−0.0041 (9)
C11	0.0329 (9)	0.0438 (11)	0.0432 (11)	−0.0032 (8)	0.0259 (9)	−0.0038 (9)
C12	0.0344 (9)	0.0425 (11)	0.0439 (11)	−0.0014 (8)	0.0254 (9)	−0.0038 (9)
C13	0.0373 (10)	0.0471 (12)	0.0443 (11)	−0.0038 (9)	0.0247 (9)	−0.0031 (9)
C14	0.0500 (13)	0.0471 (13)	0.0534 (13)	0.0009 (10)	0.0302 (11)	0.0039 (11)
C15	0.0490 (13)	0.0571 (15)	0.0614 (15)	0.0134 (12)	0.0291 (12)	0.0067 (12)
C16	0.0410 (13)	0.0770 (19)	0.083 (2)	0.0117 (12)	0.0384 (14)	0.0138 (15)
C17	0.0417 (12)	0.0630 (15)	0.0771 (18)	0.0039 (11)	0.0402 (13)	0.0134 (14)

### Geometric parameters (Å, °)

**Table:**

Br1—C10	1.8867 (19)	C5—H5A	0.92 (3)
O1—N2	1.376 (2)	C7—C8	1.424 (3)
O1—C7	1.431 (2)	C8—C9	1.477 (3)
O2—C7	1.193 (3)	C9—C10	1.480 (3)
O3—C9	1.213 (3)	C10—C11	1.335 (3)
N1—N2	1.303 (2)	C11—C12	1.462 (3)
N1—C8	1.359 (2)	C11—H11A	0.93 (2)
N1—C6	1.450 (2)	C12—C13	1.396 (3)
C1—C6	1.377 (3)	C12—C17	1.405 (3)
C1—C2	1.380 (3)	C13—C14	1.380 (3)
C1—H1A	0.93 (3)	C13—H13A	0.94 (2)
C2—C3	1.374 (4)	C14—C15	1.376 (4)
C2—H2A	0.87 (3)	C14—H14A	0.96 (3)
C3—C4	1.373 (4)	C15—C16	1.368 (4)
C3—H3A	0.93 (3)	C15—H15A	0.93 (3)
C4—C5	1.387 (3)	C16—C17	1.377 (4)
C4—H4A	0.92 (3)	C16—H16A	0.95 (3)
C5—C6	1.373 (3)	C17—H17A	0.97 (3)
			
N2—O1—C7	111.17 (15)	C7—C8—C9	131.22 (18)
N2—N1—C8	115.34 (16)	O3—C9—C8	118.59 (19)
N2—N1—C6	117.16 (16)	O3—C9—C10	122.82 (19)
C8—N1—C6	127.29 (16)	C8—C9—C10	118.59 (17)
N1—N2—O1	104.55 (15)	C11—C10—C9	122.08 (18)
C6—C1—C2	118.1 (2)	C11—C10—Br1	125.70 (16)
C6—C1—H1A	119.2 (16)	C9—C10—Br1	112.17 (15)
C2—C1—H1A	122.8 (16)	C10—C11—C12	134.49 (19)
C3—C2—C1	120.7 (2)	C10—C11—H11A	112.3 (13)
C3—C2—H2A	122.1 (19)	C12—C11—H11A	113.2 (14)
C1—C2—H2A	117.2 (19)	C13—C12—C17	117.8 (2)
C4—C3—C2	120.0 (2)	C13—C12—C11	116.55 (19)
C4—C3—H3A	121.3 (17)	C17—C12—C11	125.6 (2)
C2—C3—H3A	118.7 (17)	C14—C13—C12	121.4 (2)
C3—C4—C5	120.6 (2)	C14—C13—H13A	122.0 (15)
C3—C4—H4A	120.5 (18)	C12—C13—H13A	116.5 (14)
C5—C4—H4A	118.9 (18)	C15—C14—C13	119.7 (2)
C6—C5—C4	118.0 (2)	C15—C14—H14A	121.0 (17)
C6—C5—H5A	118.9 (15)	C13—C14—H14A	119.4 (17)
C4—C5—H5A	123.2 (15)	C16—C15—C14	119.9 (2)
C5—C6—C1	122.6 (2)	C16—C15—H15A	119.6 (19)
C5—C6—N1	119.33 (18)	C14—C15—H15A	120.4 (19)
C1—C6—N1	118.08 (18)	C15—C16—C17	121.5 (2)
O2—C7—C8	136.9 (2)	C15—C16—H16A	120 (2)
O2—C7—O1	120.12 (19)	C17—C16—H16A	118 (2)
C8—C7—O1	102.93 (16)	C16—C17—C12	119.7 (3)
N1—C8—C7	105.97 (17)	C16—C17—H17A	120.5 (17)
N1—C8—C9	121.14 (17)	C12—C17—H17A	119.8 (17)
			
C8—N1—N2—O1	1.9 (2)	O2—C7—C8—C9	−14.3 (4)
C6—N1—N2—O1	176.99 (14)	O1—C7—C8—C9	164.64 (19)
C7—O1—N2—N1	−2.04 (18)	N1—C8—C9—O3	34.8 (3)
C6—C1—C2—C3	−0.3 (4)	C7—C8—C9—O3	−128.2 (2)
C1—C2—C3—C4	0.1 (5)	N1—C8—C9—C10	−144.59 (19)
C2—C3—C4—C5	−0.1 (5)	C7—C8—C9—C10	52.3 (3)
C3—C4—C5—C6	0.3 (4)	O3—C9—C10—C11	−169.2 (2)
C4—C5—C6—C1	−0.6 (4)	C8—C9—C10—C11	10.2 (3)
C4—C5—C6—N1	179.9 (2)	O3—C9—C10—Br1	8.3 (3)
C2—C1—C6—C5	0.5 (4)	C8—C9—C10—Br1	−172.26 (15)
C2—C1—C6—N1	−180.0 (2)	C9—C10—C11—C12	179.9 (2)
N2—N1—C6—C5	57.7 (3)	Br1—C10—C11—C12	2.7 (4)
C8—N1—C6—C5	−127.9 (2)	C10—C11—C12—C13	−171.1 (2)
N2—N1—C6—C1	−121.9 (2)	C10—C11—C12—C17	11.2 (4)
C8—N1—C6—C1	52.6 (3)	C17—C12—C13—C14	−1.0 (4)
N2—O1—C7—O2	−179.32 (17)	C11—C12—C13—C14	−178.9 (2)
N2—O1—C7—C8	1.49 (18)	C12—C13—C14—C15	0.5 (4)
N2—N1—C8—C7	−1.0 (2)	C13—C14—C15—C16	0.0 (4)
C6—N1—C8—C7	−175.51 (16)	C14—C15—C16—C17	−0.1 (5)
N2—N1—C8—C9	−167.81 (17)	C15—C16—C17—C12	−0.4 (5)
C6—N1—C8—C9	17.6 (3)	C13—C12—C17—C16	0.9 (4)
O2—C7—C8—N1	−179.3 (2)	C11—C12—C17—C16	178.6 (3)
O1—C7—C8—N1	−0.36 (18)		

### Hydrogen-bond geometry (Å, °)

**Table:**

Cg1 is the centroid of the C12–C17 benzene ring.

**Table:**

*D*—H···*A*	*D*—H	H···*A*	*D*···*A*	*D*—H···*A*
C2—H2A···O3^i^	0.87 (3)	2.58 (3)	3.126 (3)	122 (2)
C3—H3A···O3^i^	0.93 (3)	2.53 (3)	3.140 (4)	124 (2)
C5—H5A···O2^ii^	0.92 (3)	2.47 (3)	3.388 (3)	171 (2)
C17—H17A···Br1	0.97 (3)	2.66 (3)	3.364 (3)	130 (3)
C14—H14A···Cg1^iii^	0.96 (3)	2.86 (3)	3.639 (3)	139 (2)

Symmetry codes: (i) −*x*+1, *y*−1/2, −*z*+1/2; (ii) −*x*+1, −*y*+2, −*z*+1; (iii) −*x*+2, *y*−1/2, −*z*+3/2.
